# Another Remedy for Consumption

**Published:** 1887-07

**Authors:** 


### *ANOTHER REMEDY FOR CONSUMPTION.*

Sommerbrodt urgently advocates the exhibition of creasote in phthisis, and claims that the results he obtained in five thousand cases treated with this drug have been surprisingly satisfactory. He gives capsules containing $\frac{3}{4}$ grain of creasote and $\frac{1}{3}$ grain of balsam of tolu, of which he orders one to be taken the first day, two the second day, and three on the eighth day, in water, one after each meal. The second week he distributes in the same three periods four capsules, the third week five, and the fourth week six capsules. If the drug—which is stated to be generally the case—is well borne, six capsules are ordered daily for two months in succession. After discontinuing the capsules for a month or so, this medication may, if deemed necessary, be continued for one year. Patients have taken as much as six hundred to two thousand capsules without interruption. The usual hygienic and dietetic rules, of course, are to be strictly enforced besides. Save some eructation and occasional increase of the menstrual flow, creasote did not give rise to any secondary effects. The system becomes quickly and surprisingly accustomed to the drug. Very advanced forms of phthisis were not influenced by the treatment, while the initial phases, such as small infiltrations or catarrh, of the apex combined with hæmoptysis, showed very pronounced signs of improvement. Young individuals, particularly, were benefited by this treatment. The author obtained the same beneficent effects from creasote in the treatment of scrofulosis. The cough, even if caused by a copious bronchial secretion, yields promptly to this medication, and fever and sweats become likewise reduced. In numerous cases could the physical signs be no longer detected.—*Berlin. Klin. Wochen.*, No. 15, 1887.

